# Engineering Tendon: Scaffolds, Bioreactors, and Models of Regeneration

**DOI:** 10.1155/2016/3919030

**Published:** 2015-12-28

**Authors:** Daniel W. Youngstrom, Jennifer G. Barrett

**Affiliations:** Department of Large Animal Clinical Sciences, Marion duPont Scott Equine Medical Center, Virginia Tech, 17690 Old Waterford Road, Leesburg, VA 20176, USA

## Abstract

Tendons bridge muscle and bone, translating forces to the skeleton and increasing the safety and efficiency of locomotion. When tendons fail or degenerate, there are no effective pharmacological interventions. The lack of available options to treat damaged tendons has created a need to better understand and improve the repair process, particularly when suitable autologous donor tissue is unavailable for transplantation. Cells within tendon dynamically react to loading conditions and undergo phenotypic changes in response to mechanobiological stimuli. Tenocytes respond to ultrastructural topography and mechanical deformation via a complex set of behaviors involving force-sensitive membrane receptor activity, changes in cytoskeletal contractility, and transcriptional regulation. Effective *ex vivo* model systems are needed to emulate the native environment of a tissue and to translate cell-matrix forces with high fidelity. While early bioreactor designs have greatly expanded our knowledge of mechanotransduction, traditional scaffolds do not fully model the topography, composition, and mechanical properties of native tendon. Decellularized tendon is an ideal scaffold for cultivating replacement tissue and modeling tendon regeneration. Decellularized tendon scaffolds (DTS) possess high clinical relevance, faithfully translate forces to the cellular scale, and have bulk material properties that match natural tissue. This review summarizes progress in tendon tissue engineering, with a focus on DTS and bioreactor systems.

## 1. Introduction

Tendons connect muscle to bone, functioning in force translation and energy storage during movement [1]. Up to 80% of the dry mass of tendon is fibrillar collagen, and the specific mechanical properties of tendon are largely the result of type-I collagen organization within the extracellular matrix (ECM) [2]. Three *α*-helical molecular [Gly-x-y]_*n*_ collagen strands form the triple helical foundation of tendon structure: these are quarter-staggered into banded microfibrils that are hierarchically arranged into secondary and tertiary bundles [3, 4]. Collagen expression within tendon is regulated by a number of molecules including the transcription factor scleraxis [5, 6]. Procollagen molecules undergo posttranslational modifications and their assembly is regulated by molecular chaperones [7, 8]. Type-III collagen is an important minority component of tendon ECM, and its elevated presence is associated with decreased fiber diameter [9] and postinjury repair [10]. Type-V collagen is also present in the core of fibrils and contributes to the structural arrangement of ECM more than its mechanical properties [11].

Partially a result of its characteristically low cellularity relative to other tissues, tendon matrix was once thought to be inert of metabolic activity [12]. However, much like the classic frameworks of bone and soft tissue remodeling (Wolff's Law and Davis' Law, resp.) [13], tendons dynamically respond to loading events [14], with different tendons exhibiting variations by function [15]. Tenocytes are the terminally differentiated cells resident to tendon and are generally responsible for maintaining ECM homeostasis. Tenocytes align along the proximal-distal axis parallel to fiber direction, extending projections deep into their extracellular environment and maintaining cell-cell connectivity through cadherin-11 junctions [16]. Matrix metalloproteinases (MMPs) degrade ECM and include secreted gelatinases (MMP-2 and MMP-9), collagenases (MMP-1, MMP-8, and MMP-13), and stromelysins (MMP-3, MMP-10, and MMP-11) among others [17, 18]. Dysregulation of these enzymes is a trait of degeneration: MMP-2, MMP-3, MMP-14, and MMP-19 are significantly upregulated in human tendinopathy [19]. Healthy tenocytes deposit collagen to counteract this degradation [20]. The metabolic activities of tendon cells differ from tendon to tendon, and it is uncertain if this is a developmental trait or the result of adaptation to a specific mechanical environment [21].

Glycosaminoglycans (GAGs) are linear polysaccharide chains covalently bonded to proteoglycan cores within the ECM [3]. GAGs are important extracellular regulators, assisting in the lateral aggregation of type-I collagen [22] and water homeostasis [23], altering tissue biomechanics, and resisting compression [24, 25]. GAGs also nonspecifically bind growth factors, giving the ECM additional regulatory properties [26]. GAG content must be carefully maintained, as over- or underproduction has negative effects. In addition to remodeling collagen, tenocytes are responsible for managing proteoglycan turnover. In bovine deep flexor tendon explants, large proteoglycans have half-lives of approximately two days [27]. This is comparatively rapid for ECM proteins, particularly as carbon turnover virtually ceases in humans after adolescence [28]. Changes in proteoglycan turnover likely come before detectable structural alterations [29]. Increased GAG content has been found in diseased tendons [30], while GAG digestion lowers viscoelasticity [31, 32]. GAG exposure in damaged tendon may also be an irritant related to the pain of tendinopathy [33]. Thus GAGs are inexorably tied to tendon health.

The mechanical behavior of tendon and other materials is frequently represented in a stress/strain curve, which plots elongation versus force per cross-sectional area [34]. The classical response demonstrates an initial toe region, a linear-elastic region, a plastic deformation region, and a point of failure. This model fits the deformation properties of ligament and tendon, and parameters of this relationship change during injury and aging [35, 36], ultimately altering cellular behavior [37]. As a complex viscoelastic biomaterial, tendons also exhibit force-relaxation, creep, and hysteresis [38]. While several natural and synthetic scaffolds are available as alternatives, the easiest way to replicate the totality of the properties of native tendon is to use decellularized tendon as a substrate. The scientific and clinical value of decellularized tendon scaffolds (DTS) may be further enhanced using cultivation tools such as bioreactors.

In the context of tissue engineering, the term “bioreactor” describes any (typically* in vitro*) culture system that not only sustains the life of cells/tissues outside the body but also enriches the cellular environment with dynamic stimuli designed to promote a particular phenotype. Soft tissue bioreactors are still in their infancy, with few biomimetic scaffold systems available. Even rarer are systems incorporating naturally derived scaffolds, despite the long history of tendon allografts in clinical practice. DTS remains the only option possessing the (1) structure, (2) composition, and (3) biomechanical properties of native tendon. This review will outline historical and current developments in the area of decellularized tendon scaffolds and their application in bioreactor systems.

## 2. Tendon Decellularization

Tendon autotransplantation developed as a discipline in response to unprecedented numbers of combat casualties in the wake of World War I [39], but it was not until the mid-1950s that allografts [40] and artificial tendons [41] first entered trials. Tendon healing after trauma is ordinarily accomplished by a combination of cells both intrinsic and extrinsic to the tendon mid-substance [42], but it was uncertain how tendon grafts integrated with the host, and in turn how that process might be improved. Tendon scaffold was first used for the purpose of basic cell biological research in 1986, when rabbit quadriceps patellar tendon autografts were flash-frozen in liquid nitrogen and used for anterior cruciate ligament (ACL) reconstruction in order to demonstrate the donor origin of repopulating cells [43]. While not “decellularized,” these constructs were devoid of live donor cells, allowing the first observations into the active role of cells in tendon homeostasis. Infiltration by peripheral cells was found to be insufficient to restore full biomechanical functionality. The failure strain of freeze-killed medial collateral ligaments (MCLs) orthotopically transplanted in a rabbit model decreased by 25% versus their fresh cell-containing counterparts nearly one year after the operation [44]. Cells quickly became the focus of tendon reconstructive research after this discovery [45]. Achilles tendon prostheses containing autologous MSCs dramatically enhanced gap defect healing in rabbits [46]. Since that time, a tissue engineering approach combining cells and scaffolds has been widely explored in effort to enhance tendon regeneration [47].

Decellularization protocols were invented in order to prevent the immunogenicity seen following anterior cruciate ligament repairs with freeze-dried allograft of xenograft [48]. Though some aspects of collagen structure are not conserved, the primary agents responsible for inciting tendon graft rejection are donor cells [49]. Decellularization is necessary to promote an M2 decision in the “fight or fix [50]” host macrophage response [51]. The predominance of type-I collagen in tendon ECM poses a particularly low immunological risk once cells are removed [52]. Chloroform-methanol extraction was the first chemical decellularization technique to achieve widespread use [53, 54]. Since that time, techniques for cell removal have been incrementally improved, with increasing emphasis on tissue engineering.

Modern decellularization protocols most commonly apply detergents to solubilize cell debris. Detergents can disrupt collagen banding and mechanical properties [55], so a balance must be found in removing cells but preserving ECM. A limited number of systematic detergent decellularization protocols exist in the literature, and all use surfactants such as sodium dodecyl sulfate (SDS) and 4-octylphenol polyethoxylate (Triton X-100) or the organophosphorus solvent tri(*n*-butyl)phosphate (TnBP). Cartmell and Dunn compared the effects of Triton X-100, TnBP, and SDS at 0.5–2.0% concentration on rat tail tendons for 12–48 hours and found that 1% SDS for 24 hours or 1% TnBP for 48 hours most effectively removed cells and maintained histological and biomechanical features of normal tendon [56]. Woods and Gratzer observed that, of two-step detergent protocols involving 48-hour incubation in 1% Triton X-100 followed by another 48 hours in 1% solutions of SDS, Triton X-100, or TnBP, the Triton-SDS combination worked best for 300 *μ*m thick canine Achilles tendon ribbons [57]. Interestingly, the same group discovered that a different combination (Triton-TnBP) was more effective at porcine ACL decellularization [58]. Xing and colleagues compared 24-hour incubations of 1% Triton X-100, 0.5% SDS, 1% TnBP, 1% Triton X-100 with 0.5% SDS, 1% TnBP with 0.5% SDS, and 1% TnBP with 1% SDS to decellularize rabbit semitendinosus and flexor digitorum tendons and found 1% Triton X-100 with 0.5% SDS to be most effective in removing cells without damaging mechanical strength [59]. Deeken et al. compared 1% Triton X-100 and 1% Triton X-100 with 1% TnBP, 2% TnBP, 1% TnBP, 1% SDS, and 2% SDS and found 1% TnBP to be most effective at decellularizing porcine diaphragm tendon [60].

Our group was the first to use an equine tendon scaffold for tissue engineering applications. This model is advantageous due to the size, availability, low vascularity, and high mechanical strength of equine tendons relative to other species. Briefly, we compared the effect of 1% TnBP, 1% SDS, 2% SDS, and 0.5% Triton X-100 with 0.5% SDS with a detergent-free group on 400 *μ*m thick equine flexor digitorum superficialis tendon (FDST) ribbons, finding that 2% SDS in combination with other methods provided a nearly cell-free, biomechanically robust, and biocompatible scaffold material [61, 62]. Flexor tendon allografts needed for hand reconstruction typically fall within the range of 2–7 cm [63], and decellularized equine FDST ribbons may prove to be ideally suited for this application. Burk et al. later decellularized full-thickness equine FDST samples using 48 hours of 1% Triton X-100 incubation in combination with freeze/thaw cycles [64]. On the human side, Pridgen et al. compared 1% Triton X-100, 1% TnBP, 1% SDS, and 0.1% SDS, and 0.1% SDS was sufficient to decellularize FDST and flexor digitorum profundus tendon (FDPT) [65]. Hammer et al. decellularized human iliotibial tract using 1% SDS, noting incomplete DNA removal (44.7% residual) and native tensile properties, and incorrectly stating that theirs was the first study to compare matched native and decellularized tendon samples [66]. Other techniques such as freeze/thaw cycles [67], hypotonic solutions [68], nucleases [69], oxidizing agents [70], and irradiation [71] have also been used on tendon, alone or in combinations with detergents.

Human FDPTs, decellularized in SDS and implanted into outbred rats, had decreased immunogenicity and improved mechanical properties versus nondecellularized counterparts [72]. A similar result was seen in 2% SDS-decellularized rat Achilles tendons [73]. Of the commercial tissue augmentation materials surgically used for tendon reconstruction, those that are based on natural collagen matrices are stronger and better retain sutures [74, 75]. However, the most common products, such as Graftjacket RTM (Kinetic Concepts, Inc.), Allopatch HD (Musculoskeletal Transplant Foundation), and TissueMend Soft Tissue Repair Matrix (Stryker), are (human and human and bovine, resp.) dermal allografts and lack the standalone biomechanical properties of tendon. Full coverage of nontendinous ECM scaffolds is beyond the scope of this review, but some groups have experimented with surface modification or reinforcement techniques to add mechanical strength to ECM sheets [76–78], while others embrace the sacrificial nature of weak but rapidly remodeled scaffolds as encouraging* de novo* tissue growth. As previously stated, tendon grafts lacking live cells are in widespread use for ACL replacement but do not fully restore native function [79]. It can take three years or longer for allografts to reach peak integration, which nevertheless remains incomplete and weak compared to healthy tissue [80]. While some groups are experimenting with functionalization or composite techniques [81], another potential solution to this problem is to provide a cell population that remodels and strengthens the scaffold over time.

Reseeded decellularized tendon scaffolds have demonstrated strong potential as graft materials [82], but animal testing is required [83]. Multilayer composites of decellularized canine infraspinatus tendons laden with rabbit bone marrow-derived MSCs remained vital, began to express tenomodulin, and altered collagen and matrix metalloproteinase activity [84]. Rabbit rotator cuffs repaired with matrix performed better after 8 weeks when seeded with tendon cells [85]. Human FDPT decellularized in 0.1% SDS and peracetic acid then seeded with adipose-derived MSCs implanted subcutaneously in nude mice remained healthy and viable for one month [86]. Reseeded Triton X-100-treated rat Achilles tendons were stronger and better organized after 24 hours in a surgical replacement model [87]. However, cell-laden tendon constructs cultured* ex vivo* without mechanical manipulation actively degrade their scaffolds, necessitating culture techniques that fulfill the need for cells to experience stimulation [88].

## 3. Tendon Bioreactors

*In vitro* cell culture was pioneered by Carrel at the turn of the 20th century. By nourishing tissue explants in plasma enriched with various animal-derived extracts, Carrel extended the life expectancy and proliferative capacity of cells in culture from weeks to months [89, 90]. Realizing that diffusion-limited nutrient flow hindered not only the size of tissue explants but also the vitality of transplant organs, Carrel, in collaboration with Lingbergh, coinvented “an apparatus for the culture of whole organs” [91], the first perfusion bioreactor.

Musculoskeletal loading plays an important role in tissue homeostasis, a fact that gained increasing attention as humans established technologies facilitating spaceflight. Astronauts experience reversible bone demineralization [92], which is attributed to attenuated bone formation but retained bone resorption while in orbit [93]. It was recognized that rat bone marrow cells lose osteogenic potential when unloaded* in vivo* [94] but have improved osteogenic potential when cultured on flexible-bottom culture dishes subject to 1 Hz deformation cycles [95]. As gravitational and locomotive forces have been essential evolutionary conditions, many tissues experience similar phenomena. ECM forces and cell shape result in fate decisions including differentiation and apoptosis [96], and transplanting cells can induce progenitor cell differentiation into tissues other than their source of origin [97]. Modern musculoskeletal bioreactors are direct decedents of these discoveries and attempt to manipulate these effects. Deformation protocols are most efficacious when they resemble the* in vivo* environment [98], as cells respond differently to deformations induced by compression versus tension [99].

Elastic deformation of tendon occurs at the 100 *μ*m level by straightening of fibrillar crimp and then at the 10–15 nm level by molecular elongation of collagen helices [100]. Sensation of these cell-scale forces, such as through integrin-activated MAP kinase and NF-*κ*B signaling or by stress-responsive gene enhancers, is essential to proper tenocyte phenotype [101]. Stretch also alters the availability and rate of ECM binding domains involved in assembly and degradation [102]. Tendons deprived of mechanical stimulation* in vitro* experience profound negative alterations including changes in anatomical size, a near complete loss of biomechanical function, hypercellularity, and decreased ECM alignment [103]. This occurs even in tendons frozen* in situ* [104]: a treatment which may in fact exacerbate ECM catabolism through proteolytic enzyme release. Cells exhibit sensitivity to subtle changes in their mechanical environment, with small deformations frequently leading to an anabolic and anti-inflammatory response and large deformations leading to inflammation and ECM damage [105]. Selectivity to static versus dynamic forces of the same magnitude is also provided by differences in resistance to conformational change among components of intracellular focal adhesion complexes [106]. While other types of bioreactors such as microcarriers [107, 108], flow perfusion, and shear systems [109–112] and hydrostatic bioreactors [113] exist, this review focuses on systems involving stretching or mechanical load that attempt to model natural deformation.

Banes made pioneering advances in the musculoskeletal mechanobiology field with his invention of the Flexcell bioreactor platform in 1985 [114]. This system uses flexible-bottom circular culture wells deformed via vacuum to deliver controlled mechanical signals to cells, allowing mechanistic* in vitro* studies of tendon and ligament signaling [115]. For example, ligaments undergo atrophy when unloaded, as sensed via fibronectin-specific integrin *α*_5_ *β*_1_ and other mechanisms [116]. Cell stretching not only encourages tissue anabolism but also results in cell-mediated ECM recomposition. Primary human MCL cells increasingly express type-III collagen under 7.5% but not 5% strain in a Flexcell bioreactor at 1 Hz for 16–25 hours [117]. This effect is not observed in cells derived from synovium [118]. Human tendon fibroblasts secrete the growth factors TGF-*β*, bFGF, and PDGF in response to stretch on the timescale of hours [119]. Flexcell bioreactors can be programmed to conform to virtually any waveform desired, but the resulting deformation is only precise in the center of each well [120], even after careful calibration [121]. A slightly more uniform approach is to use a uniaxial stretch system to deform rectangular silicon dishes using a stepper motor, the use of which demonstrated TGF-*β*1-mediated expression of type-I and type-III collagen in human ACL fibroblasts [122].

Scaffold-free flexible plastic may be considered the first generation of tendon/ligament bioreactors. While they represent a tremendous increase in complexity versus traditional monolayer culture, they are far from modeling the* in vivo* environment. Second-generation bioreactors are based on three-dimensional scaffolds but do not mimic the alignment or function of native tendon. As the principle component of tendons and ligaments, type-I collagen is the most common base, but the lack of hierarchical organization in collagen hydrogels makes them structurally inferior for translational use [123]. Tendon cells do, however, interact with collagen gels, resulting in changes in phenotype [124]. Butler's group has developed functionalized type-I collagen-based sponge constructs for tendon repair that can withstand forces experienced in rabbits [125–127]. Rabbit bone marrow-derived MSCs embedded in these sponges established maximum stiffness when cultured at 2.4% strain at 1 Hz for 50 minutes per day [128]. Human tenocytes cultured in reconstituted rat tail collagen stretched at 5% strain at 1 Hz for 48 hours resulted in differential expression of several proteases and matrix proteins, as well as TGF*β* activation [129]. Many natural but isotropic materials used for surgical augmentation have been used in bioreactors with varying results, including porcine small intestine submucosa [130] and human umbilical veins [131], as well as designer scaffolds such as the woven hyaluronic acid-based Hyalonect [132]. Biaxial deformation is possible in these bioreactors [133], but multiaxial strain is not typically applicable to tendon physiology.

Synthetic hydrogels are also commonly implemented, and their properties are tunable [134]. A commercial human MSC line encapsulated in poly(ethylene glycol)- (PEG-) based scaffolds under 10% strain at 1 Hz for alternative 3-hour on/off periods for 21 days resulted in upregulation of tenocytic genes including collagens and tenascin-C [135]. Rabbit Achilles tendon cells in porous poly(L-lactide-co-*ε*-caprolactone) (PLCL) scaffolds under 10% strain at 0.25 Hz for 400 minutes per day for 4 weeks demonstrated enhanced proliferation and type-I collagen deposition [136]. 3D culture is critical for proper morphology, but disordered hydrogels do not deform via the same mechanisms of native ECM. Hydrogels contract and remodel and may begin to establish alignment but do not resemble tendon [137, 138]. Cultured MSC sheets deposit ECM on plastic, which can also be used as a simple matrix. One study using such a system discovered that both mechanical forces and scleraxis can independently induce tenogenesis, but their influences are synergistic and most effective when combined [139]. This strategy falls into the same technical class as hydrogels: useful but not biomimetic. Nevertheless, fundamental signaling information, such as the nature of the refractory period following periods of mechanical stimulation [140], is likely conserved. However, heterogeneity in the structural properties of different systems complicates comparisons, such as cell-scale felt strain.

Third-generation bioreactors implement aligned scaffolds, natural or synthetic, and more faithfully recapitulate tendon structure and alignment but are not biomechanically functional as standalone replacement tendons. Rat MCL cells aligned on collagen-coated polydimethylsiloxane (PDMS) scaffolds demonstrated increased cell-cell calcium signaling sensitivity versus nonaligned controls [141]. Another option is to use synthetic nano/microfibers. These have better structural similarity to tendon, induce spindle morphology, and when sized correctly provide structural cues that aid in tenogenesis [142]. Human ligament fibroblasts cultured on electrospun polyurethane nanofibers increased ECM production in response to 5% strain at 0.2 Hz in a modified Flexcell bioreactor [143]. Cardwell et al. cultured C3H10T1/2 MSCs on electrospun poly(ester urethane) urea fibers, loaded daily by 4% strain at 0.5 Hz for 30 minutes, witnessing alignment and a tenocytic gene expression profile [144]. Silk scaffolds are widely used by Moreau and his collaborators as a platform for MSC differentiation toward ligament [145]. Aligned, cross-linked collagen fibers most closely approximate the structural and mechanical properties of native tendon. In a recent study by Qiu et al., human bone marrow-derived MSCs cultured under 10% strain at 1 Hz for alternating 3-hour on/off cycles for 14 days proliferated more and expressed greater amounts of scleraxis, tenascin-C, and collagens than their static counterparts [146].

The most holistic tendon bioreactors use decellularized tendon matrix as a scaffold subject to cyclic strain. These are the fourth-generation tendon bioreactors, which replicate not only the mechanical environment of native tendon but also the complex ultrastructure, composition, and biomechanical properties. There are currently only four principle investigators using this technique: Chang of Stanford University, Van Dyke of Wake Forest University, Barrett at Virginia Tech, and Zhao at Mayo Clinic. Chang's group released back-to-back “Tissue Engineering: Part A” papers in 2010, characterizing the response of rabbit and human flexor tendon constructs cultured in Ligagen L30-series axial bioreactors. P1 rabbit FDPT-derived cells cultured on decellularized FDPT increased construct elastic modulus and ultimate tensile strength in response to cyclic 1.25 N strains at 0.0167 Hz for alternating one-hour periods over 5 days [147]. In a similar study with matched human FDST and EDST sets (P4) with 0.625 N–2.5 N strains over 3–8 days, the investigators observed time-dependent improvements in biomechanical properties but no differences between groups of different load magnitudes [148]. Constructs then degraded after two days of disuse. Van Dyke's group released a 2013 paper using allogeneic chicken FDPT constructs (P4) exposed to 5% strain at 1 Hz for one hour per day for 7 days in the same Ligagen system. Cells preserved construct mechanical properties versus unseeded controls, but no significant changes in mRNA profiles were seen resulting from strain [149]. Our group used a custom bioreactor to characterize amplitude-dependent gene expression profiles of P2 horse bone marrow MSCs, finding that 3% strain at 0.33 Hz for one hour per day increased ultimate tensile strength and induced high expression of scleraxis, type-I collagen, and proteoglycans [62]. Qin et al. released the results of a similar study after our 2015 Journal of Orthopaedic Research article was published, reaching analogous conclusions in a canine Achilles tendon model [150].

## 4. Applications

Bioreactors are used for (1) basic pathway studies, (2) growing replacement tissues, (3) maintenance of organ vitality* ex vivo*, and (4) priming therapeutic cells prior to cell transplantation. The first tendon tissue bioreactor study was conducted by Arnoczky's group, when Hannafin et al. found that canine FDPT maintained its mechanical properties* ex vivo* for 4 weeks while unloaded controls degraded [151]. Lavagnino et al. later performed multiple sets of experiments to determine that frequency and amplitude resulted in dose-dependent increases in MMP-1 expression in rat tail tendons [152]. This technique is still being used to elucidate pathways involved in tendon adaptation to exercise, such as collagen and IL-6 expression [153], as well as damage resulting from repetitive loading [154] and cellular maintenance of biomechanical properties [155].

Living animals also provide valuable information for* in vitro* bioreactors. For example, following a single loading episode in rat Achilles tendons, gene expression returns to baseline after one day [156], but as little as 5 minutes of loading over 4 days is enough to improve mechanical properties [157]. While the use of animals cannot be eliminated, bioreactors reduce the necessity of laboratory animal experiments consistent with the 3Rs [158], namely, by reducing the numbers needed (by harvesting tissues at necropsy from unrelated studies for bioreactor use) and replacing them with comparable methods (synthetic scaffolds). In addition to tendon/ligament, cyclic-strain bioreactors are excellent platforms for cultivating muscle material, such as for the repair of critical-size volumetric defects [159, 160] or even artificial meat [161].

The practice of cell therapy in human and animal medicine has witnessed a recent surge in popularity and commercial viability [162]. Bioreactors will assist in gathering necessary efficacy data for these procedures. Furthermore, the decision of cell source, which influences differentiation capacity [163], is one such application of these systems. Indeed, one aim of our current work is to use our bioreactor to provide standardized* in vitro* comparisons on different commonly used stem cell sources for tendon cell therapy.

Uncertainty concerning the long-term vitality of transplanted cells and their relationship to the host is a challenge and opportunity for future development within the field. As graft size increases, so does its need for a relationship with the surrounding vasculature. Perhaps, depending on the state of niche factors in tendon and the surrounding environment, neovascularization and/or infiltration of host cells extrinsic to the graft may result in deterioration rather than strengthening of the tissue [164]. Designing grafts that avoid inflammatory damage [165] and promote a healthy graft-host relationship is essential. The identities of cells participating in tendon regeneration and the molecular pathways coordinating this behavior remain undefined [166]. Moving forward, improved* in vitro* models will likely help answer these unanswered questions.

## 5. Summary

Tendons are dynamic tissues, and tendon pathologies significantly reduce quality of life. There are no effective pharmacological therapies currently available to treat tendinopathies, and should tissue replacement be necessary, donor-matched prostheses are frequently unavailable. Recent developments in* ex vivo* modeling techniques have allowed us to dissect structure/function relationships and elucidate cell-ECM interactions with unprecedented accuracy and environmental control. Bioreactors are the best available tools for developing novel regenerative treatments and cultivating functional replacement tissue. Future iterations of bioreactor technology may be even better suited to these aims and will likely be capable of replicating multitissue or transitional structures by becoming increasingly complex.
